# Freeze-Casting of Porous Biomaterials: Structure, Properties and Opportunities

**DOI:** 10.3390/ma3031913

**Published:** 2010-03-17

**Authors:** Sylvain Deville

**Affiliations:** Laboratoire de Synthèse et Fonctionnalisation des Céramiques, UMR 3080, CNRS/Saint-Gobain CREE, Cavaillon, France; E-Mail: sylvain.deville@saint-gobain.com; Tel.: +4-32-50-06-59; Fax: +4-32-50-09-04

**Keywords:** freeze-casting, porous ceramics, composites, biomaterials

## Abstract

The freeze-casting of porous materials has received a great deal of attention during the past few years. This simple process, where a material suspension is simply frozen and then sublimated, provides materials with unique porous architectures, where the porosity is almost a direct replica of the frozen solvent crystals. This review focuses on the recent results on the process and the derived porous structures with regards to the biomaterials applications. Of particular interest is the architecture of the materials and the versatility of the process, which can be readily controlled and applied to biomaterials applications. A careful control of the starting formulation and processing conditions is required to control the integrity of the structure and resulting properties. Further *in vitro* and *in vivo* investigations are required to validate the potential of this new class of porous materials.

## 1. Introduction

The freeze-casting of porous materials and ceramics in particular has received a great deal of attention during the past few years. This simple process, where a material suspension is simply frozen and then sublimated before sintering, provides materials with a unique porous architecture, where the porosity is almost a direct replica of the frozen solvent crystals. Proper control of the freezing conditions yields materials with elongated and continuous porosity along the solidification direction, a morphology very different from the typical foam-like structures mostly developed so far. This unique architecture, also yielding a unique mechanical response, has promising potential for load bearing applications, and have resulted in a great number of investigations in the past few years, in particular for ceramic materials and hybrid ceramic/polymer composites, either porous or dense. This paper is by no means intended to be an exhaustive review of the freeze-casting process; such reviews can already be found in reference [1] or [2], for example. The objective here is to highlight the recent results obtained in freeze-casting studies from the biomaterials point of view, paying particular attention to the functional requirements of such structures for biomedical applications. The main interest expressed so far lies in the processing of novel porous scaffold architectures, the most important aspects to consider are the characteristics of the porosity and its control, and the mechanical response of such scaffolds, which should be clearly assessed before investigations proceed further.

## 2. Processing Principles and Materials

**Figure 1:**
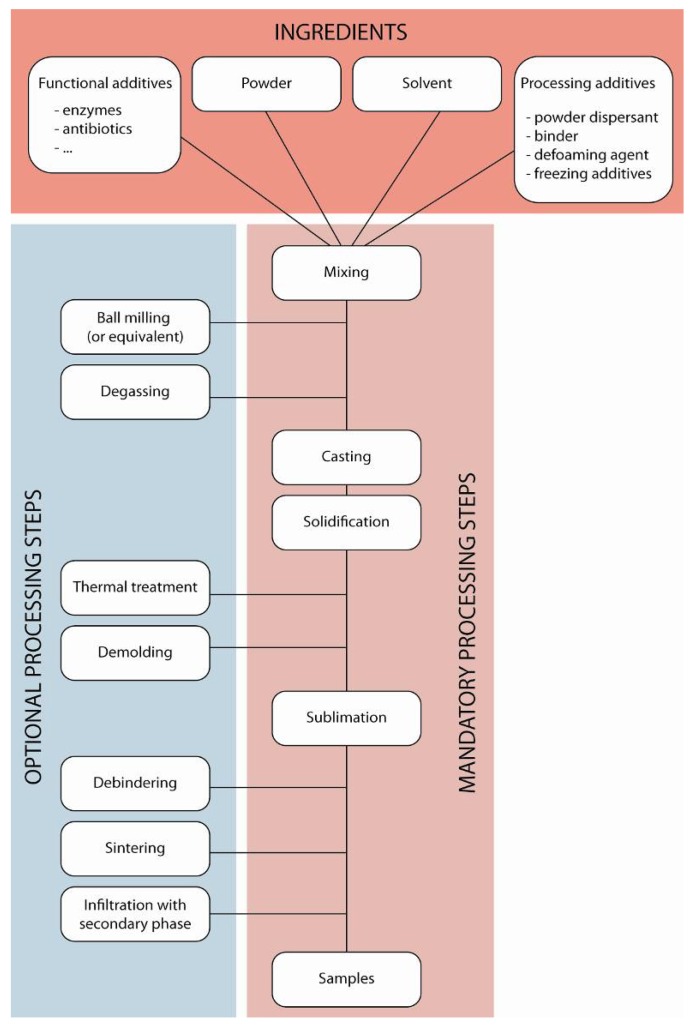
Processing steps.

The processing principles have been described in detail in previous work [1], and the main processing steps are summarized in Figure 1.

Four types of ingredients are required for the process. The ceramic powder, of course, for which certain properties are required–this point is described in more details later in the paper– must be carefully selected. The process is nevertheless extremely flexible and largely independent of the nature of the materials being used. For the solvent, various can be used, the most common being water for several reasons: convenience of use, environmental aspects, unique morphologies of the ice crystals leading to unique porosities, and friendliness with functional additives. The functional additives are the third categories of ingredients, and include for instance enzymes and antibiotics for controlled drug release. These are added with a biological functionality in mind, and can be added from the beginning of the process when no high temperature treatment steps (sintering) are required, which is the case when polymers or mixes of ceramic and polymer are used. Incorporating the functional additives from the beginning ensures their good spatial repartition in the final materials, which might be essential to ensure a progressive release into the body, for instance, in the case of antibiotics. The last category of ingredients includes all the processing additives, which are used to control the stability and dispersion of the starting suspensions, the solidification behavior (morphology of the solvent crystals) during the solidification stage and the strength and handling properties of the green body during and after sublimation.

Due to its versatility, the freeze-casting process has been applied to a broad range of materials, from polymers to ceramics and metals. The main formation mechanism of the porous structure, the rejection of particles from the growing solvent crystal, is mostly based on physical interactions, and therefore largely independent of the nature of the material that is used. A lot of the original work of freeze-casting was carried out on polymer materials, under the appellation freeze-drying or freeze-gelation [3,4,5,6,7,8,9,10,11,12,13,14,15,16,17]. Later on, attention was dedicated to ceramic materials, and in particular biomedical grade ceramics, including HAP [18,19,20,21,22,23,24,25,26,27,28,29,30,31,32], HAP-TCP [33], titania [34], bioglass [35,36], alumina [37,38], zirconia and yttria-stabilized zirconia [39,40,41,42] and alumina/zirconia composites [43]. Organic/inorganic composites can also be processed by mixing a water-soluble polymer with the ceramic powder, and numerous examples have now been described, including HAP/collagen [44], HAP/gelatine [45], HAP/alginate [46], alginate/poly (lactic-co-glycolic acid)/calcium phosphate [47], poly(a-hydroxyacid)/bioglass [48], PDLLA/bioglass [49], silicate glass/poly(L-Lactide) [50]. Finally, it is worth mentioning the possible application to titanium, using either titanium particles [51] or titanium precursor such as TiH_2_ [52,53], which are later converted to the pure metal during the heat treatment under vacuum. The freeze-casting process might eventually be a strong challenger to the current processing route for titanium foams used in biomedical systems. The use of titanium particles is nevertheless currently limited by the particle size, still very large, which imply important segregation effects and poorly defined pores, the particles and the crystals being of similar size.

## 3. Structure, Properties, and Their Control

### 3.1. Porous structure: Pore dimensions, morphologies and orientation

**Figure 2:**
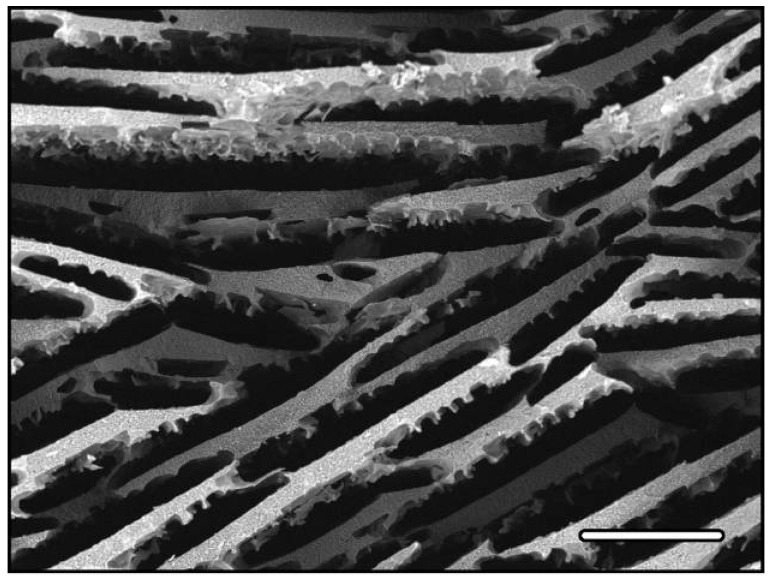
Macropores, freeze-cast alumina using water. Cross-section perpendicular to the solidification direction. Scale bar 100 μm.

The porous structure is primarily defined by the morphology of the growing solvent crystals, and secondarily by the ability of the particles to pack between the crystals, as finer particles will provide a better replica of the solvent crystals. A unique feature of the freeze-casting route is the directionality of the pores, which can be obtained under proper freezing conditions. Therefore, with very few exceptions, solidification is performed directionally, resulting in materials with macropores –or pore channels– (a few to hundreds of micrometers) running along the solidification direction (Figure 2). Ice is the most commonly used solvent and results in a lamellar structure, due to the peculiar anisotropic growth of ice Ih –the hexagonal crystal form of ice– obtained under the usual temperature and pressure conditions. The surface of the ceramic walls usually exhibits some roughness, arising from the dendritic morphologies of the solvent crystals.

**Figure 3:**
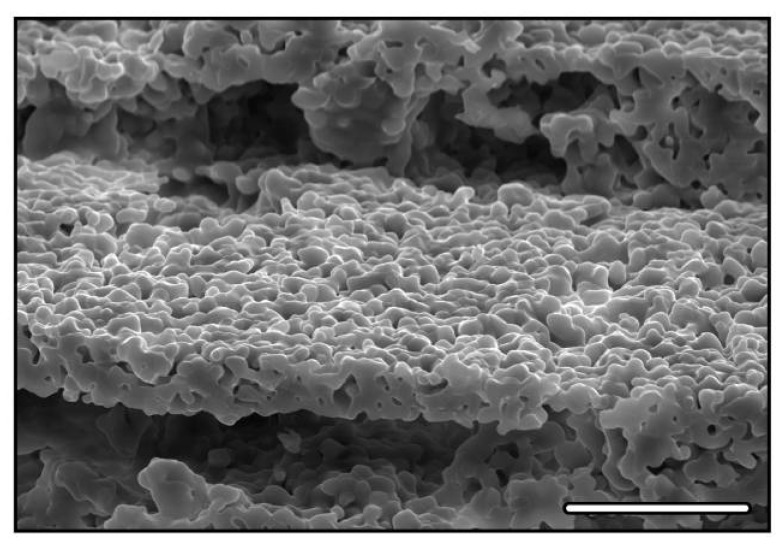
Microporosities in freeze-cast HAP. Scale bar 25 μm.

At a smaller scale, depending on the sintering conditions, submicronic pores can also be obtained [33,36] (Figure 3), such pores having been reported as beneficial for cell attachment [52].

**Figure 4:**
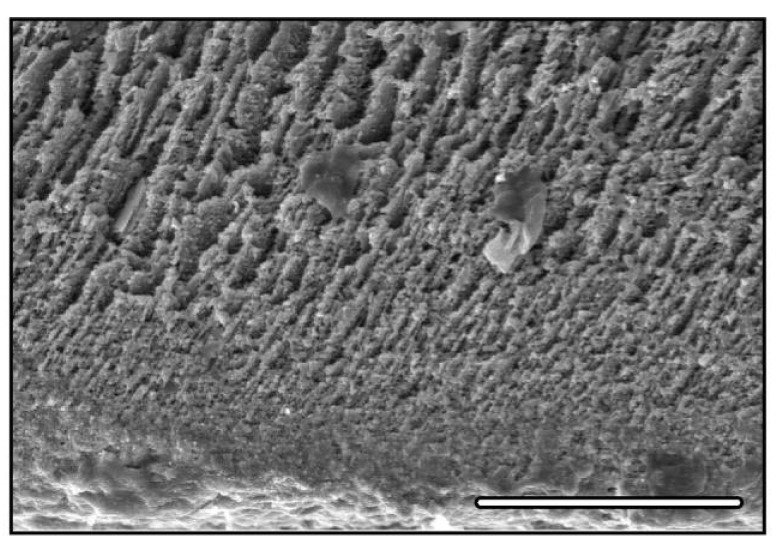
Initial gradient. Cross-section parallel to the solidification direction. The displacement of the solidification interface occurred from bottom to top. Scale bar 100 μm.

Another important feature of the structure is the systematic observation of a structural gradient in the freezing of the samples (Figure 4). Occurrence of this can be found, for instance, in figure 2 of reference [53], figure 10 of reference [54], figure 9 of reference [55], figure 12 of reference [56], figure 8 of reference [57], figure 6a-b of reference [58], and figure 2 of reference [59]. The presence of this structural gradient is explained by the initial conditions during the freezing stage, and is composed of two parts. A first dense part, with no porosity, corresponds to the formation of amorphous ice during the very first stages of solidification [2], where supercooling effects are present. A second part, with a gradient of pore size, is related to the nucleation, growth, and selection of the preferred population of crystals [60]. Being related to the initial nucleation and growth properties, the gradient cannot be avoided so far, although it might be controlled to some extent. Applying an electric field will favor the electromigration towards or away from this initial transition [59], and can partially affect the extent of this structural gradient.

**Figure 5:**
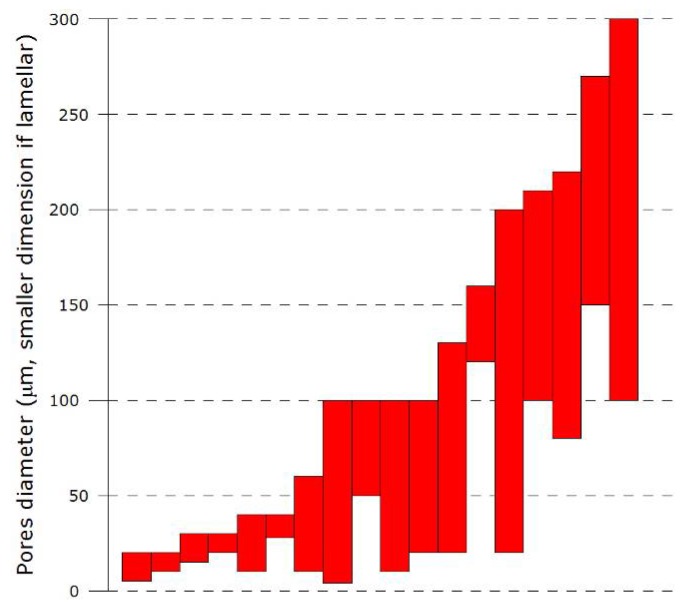
Measured or estimated pore channels size of freeze-cast ceramics, from reference [18,19,20,22,25,30,31,33,35,36,37,38,39,44,45,48,54,65,66,67]. The dimension plotted in the case of the lamellar structure corresponds to the smaller dimension of the pore, see reference [54] for further explanations. Each bar corresponds to a different study from the reference list, ranked in order of increasing maximum pore size.

The most critical parameter in the context of biomaterials scaffolds is certainly the size of the macropores, which must exceed a minimum size - under which cells penetration into the scaffolds is compromised, if not impossible. The minimum requirement is still a matter of great debate and attention, and little is known about the behavior of scaffolds with highly anisotropic architectures such as those obtained by freeze-casting. Recent results [61] report a positive influence of the presence of similar channels (500 μm in diameter, introduced through machining), in minimizing the concentration gradients of oxygen in the scaffolds, and therefore improving the initial cell seeding. In foam-like scaffolds, the critical dimension is the interconnection size between the pores: if too small, cells cannot migrate from one pore to the adjacent one. In freeze-cast materials, this concept is not relevant since the pores are essentially channels running from one side of the sample to the other. It is nevertheless now clear that a broad range of macropore dimensions can be achieved by freeze-casting, as illustrated in Figure 5.

Several techniques are available to control the pore size. The most common is a modification of the solidification rate [28,36], faster freezing resulting in smaller pores. This solution is nevertheless not very convenient to achieve large pores, since very low freezing rates are required to obtain pores of a few hundred micrometers. Control of the cooling conditions and of the suspension stability may become critical. Additional or alternative strategies have been developed, such as using other solvents instead of water, camphene [18,20,22,39,40,62,63,64,65,66] or camphene mixtures [67], naphthalene-camphor [64] being the most common choice. A thermal treatment of the solidified camphene can also be performed [18], allowing a partial recrystallization of the camphene, resulting in larger crystals and hence macropores of greater dimensions. Investigations using tert-butyl-alcool [68,69] can also be found, although EHS (Environment, Health and Safety) issues are associated with this solvent. Water-based variations such as water-dioxane [25] also seem to present promising structures. Generally speaking, any solvent other than water might present incompatibilities if functional additives are incorporated from the beginning.

A very different approach has been developed with the use of additives that affect the freezing kinetics of solidification morphologies. Numerous additives have already been tested, such as polystyrene [20,66], gelatine [19,31], sucrose, trehalose and sodium chloride [70], glycerol [25] or polyvinyl alcohol (PVA) [32]. Almost any water soluble substance is likely to affect the freezing behavior and crystal morphology; the potentialities offered here are clearly very important. Any investigation will nevertheless still be very empirical, the prediction of the additive influence on the system being extremely difficult.

**Figure 6:**
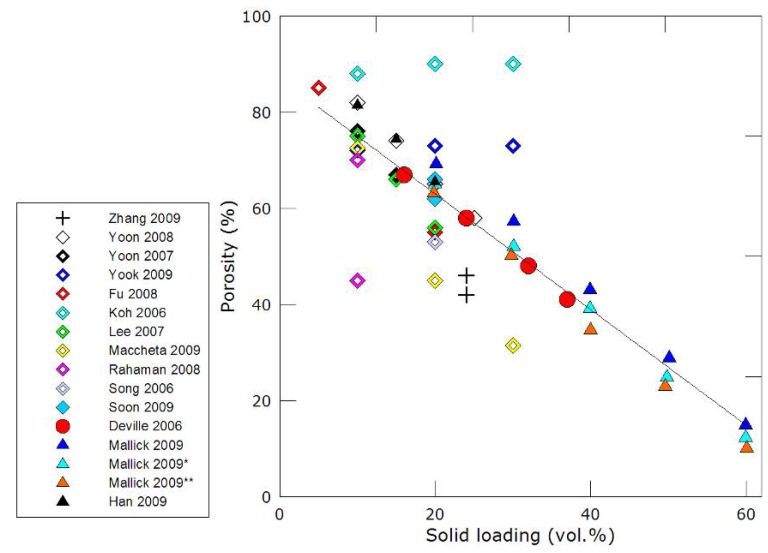
Porosity *versus* solid loading for freeze-cast porous ceramic materials [18,19,20,21,22,25,30,33,35,36,54,66,67,71].

Finally, a limited control can also be exerted through the solid loading of the starting suspension. Lower solid loading will provide some degree of increase of the macropores dimensions, although the associated loss in mechanical properties might not be acceptable. The main influence of variation in solid loading is nevertheless and obviously found in the total porosity variations (Figure 6). The total porosity will also depend on numerous additional parameters affecting the packing of particles between the solvent crystals, such as the nature of the solvent, its viscosity and surface tension, the particle size distribution and morphologies of the particles and the presence of various additives in the suspension. A large scattering of data can therefore be observed in Figure 6. There is, nevertheless, a minimum porosity amount (around 40 vol %), below which the pores are neither continuous nor interconnected anymore. The domain of interest for scaffolds is usually between 40–60 vol %, a trade-off between porosity and strength being necessary.

Natural structures are very often directional, responding to the need of carrying one or several functions along some particular directions. Wood has a continuous porous structure to carry the water where needed, bone is directional to transfer the load in an optimal way, and so forth. In freeze-casting, the porosity is defined by the characteristics of the propagation of the solidifying interface. If properly controlled, the directionality of the structure can be controlled, and several strategies have been developed to illustrate this aspect. 

Most of the time, the solidification is unidirectional; the processed materials exhibit therefore a cellular structure, highly anisotropic, where the pores are aligned along the solidification direction. Different orientation of the pore channels can nevertheless be achieved. One of the limits of unidirectional solidification is the usual lack of control of nucleation conditions: nucleation occurs randomly at the cooled surface, resulting in a random orientation of the pore channels: the porosity is continuous along the propagation direction of the solid-liquid interface, but the pore channels are randomly oriented in a plane perpendicular to the ice front propagation. Using simple, unidirectional patterns on the cooling surface [70], it is possible to manipulate the direction of the ice lamellae arising from the planar-to-lamellar transition and obtain long-range ordering with well-oriented structures on the length scale of the whole sample. More complex lamellae orientations that can be dictated by functional requirements are difficult to control by such patterning. Simple epitaxy techniques that places a mold filled with a pure solvent on a cold finger can be used to obtain circular orientations of the pore channels, which in this case exhibit an orientation perpendicular to the radial direction. The radial control of the pores parallel to the radial direction has also been obtained [33,72], by forcing the cooling in a radial direction. The solidification interface in this case is propagating from the outside toward the inside of the sample. Such structures can prove to be useful where an easy if access to the center of the processed pieces is required. Finally, gradients of porosity can also be obtained, by sequential freezing, [73] for instance. A wide variety of techniques is therefore available to achieve an isotropic or anisotropic functional response of the porous materials.

### 3.2. Mechanical properties

Surprisingly, little has been reported about the properties of freeze-cast materials. The main mechanical property of interest is usually the compressive strength, and it is often the only property assessed in the investigations. Early reports of unprecedented strength [28] have attracted a lot of attention on the process, with apparently contradictory results. Numerous studies are reporting much lower compressive strength, although a direct comparison is difficult, the materials and structures being notably different. A precise and thorough investigation of the mechanical response of freeze-cast materials is still lacking. We can nevertheless point out that the compressive strength will depend on several structural parameters:

- the nature of the material, although all ceramics have usually extremely high compressive strength values,
- the dimensions of the pores and pore channels: the smaller the channels, the higher the strength,
- the directionality and morphology of the pore channels. Unidirectionally frozen samples exhibit, without surprise, a strongly anisotropic response. The morphology of the pore channels is also of particular importance. Using camphene instead of water will result in materials with a very different porous structure, which seems to be less favorable in terms of mechanical strength.
- the integrity of the structure. This point is of particular importance, and is discussed in greater details below.

The compressive strength can be improved to some extent by forcing the formation of dense shells by melting the outside [21] of the frozen samples. This will nevertheless affect the accessibility of the structure to fluids and cells and might not be desirable for biomedical applications.

**Figure 7:**
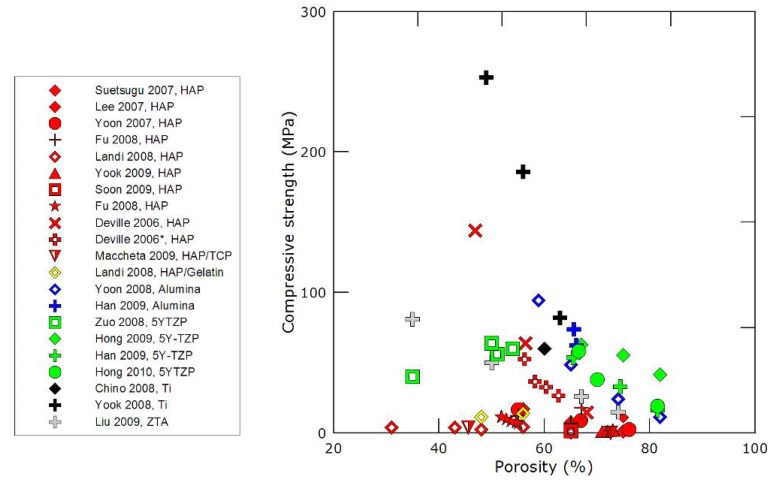
Compressive strength values *versus* material porosity percentage from literature data [18,20,22,26,27,28,30,31,33,37,39,40,41,43,45,51,54,71,76,77].

The measured values for the compressive strength of freeze-cast materials are reported in Figure 7. For the sake of interest, the values obtained for freeze-cast porous Titanium are also plotted on the graph. As mentioned above, a large scattering of the data is observed, although numerous different studies are consistently reporting high values, increasing as the porosity content is decreasing.

**Figure 8:**
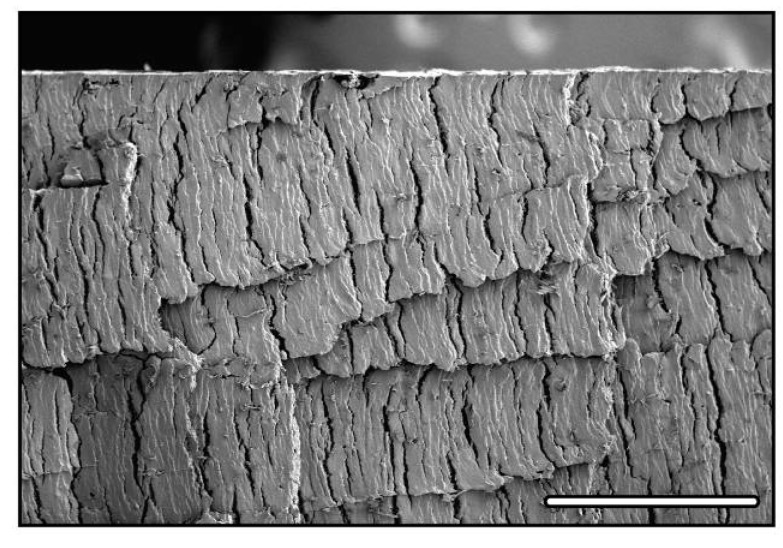
Diffusion defects in freeze-cast alumina. Cross-section parallel to the solidification direction. The displacement of the solidification interface occurred from left to right. Scale bar 700 μm.

Of particular interest are the values for HAP porous materials, of obvious interest for biomaterials applications. The majority of the values reported so far are consistently lower than the early reports, even for low porosity content (below 50%). A careful examination of the corresponding microstructures provide a first explanation to these low values: all of these low-strength samples exhibit crack-like defects in the structure (see for instance [27,45], and figure 8 of reference [74]). These defects are lying perpendicular to the solidification direction and have wrongly been attributed [20] to the sublimation stage. Recent results [75] provide an explanation to the presence and formation of these defects. These crack-like defects, illustrated in Figure 8 in the case of alumina, can be explained by the existence of instability and metastability domains in cellular solidification of colloidal suspensions. When the particles are small enough (submicronic), the diffusivity of the particles during the solidification process cannot be neglected anymore. Under unstable conditions, diffusion of the ceramic particles can proceed and nucleation of secondary ice crystals occurs. These secondary crystals result in the formation of the defects previously mentioned, which have obviously a tremendously detrimental influence over the compressive behavior of these materials. The samples with consistently low compressive strength values have been processed with powders having very low particle size, and are therefore highly susceptible to the formation of these diffusion defects, which can indeed be observed on the reported microstructures.

At this point, the following conclusions can be drawn out:

- Using small particle size is clearly not desirable here, nano is not good! The best values of compressive strength of freeze-cast samples have been obtained with particle sizes in the micrometer range, yielding defect-free structures.
- Large pore channels are often pursued, requiring very low freezing kinetics. Such conditions more often induce diffusion defects.
- The formation of these defects is directly correlated to the diffusivity of the particles. A workaround solution can nevertheless be found, for instance, by increasing the viscosity of the suspension. Interestingly, these defects have not been observed when camphene is used instead of water. The diffusivity of particles in camphene is likely to be different, and little is known about the nucleation conditions in camphene. Unfortunately, the morphology of the porous structure obtained with camphene does not seem to be optimal, with regards to the mechanical response.
- The behavior of the system with regards to the aforementioned problem will be highly dependent on the characteristics of the powder and the solvent, but also the formulation of the initial suspensions and the various additives used (surfactant, binder, *etc.*). Each system needs to be carefully assessed for its sensitivity to this phenomenon, and the formulation and processing conditions adjusted to achieve defect-free structures.

## 4. Opportunities for Biomaterials Applications

The freeze-casting process clearly offers important assets with regards to the current biomaterials requirements:

- The process is versatile: any type of ceramic or polymeric materials can be used, so that the materials composition can be adjusted to the targeted application, almost independently of its structure. For instance, the degradation rate of bioglass scaffolds can be modulated by varying the glass content of the initial formulation [50].
- The process is environmentally-friendly, in particular when water is used as a solvent.
- The compressive strength values can be extremely high, if proper control of the process is achieved, even with intrinsically weak materials such as calcium phosphate.
- The structure is highly controllable at several levels. Of particular interest is the directionality of the structure, exhibiting striking similarities with natural materials. The pore size can be adjusted to the range usually considered to be required for tissue engineering.
- The porous scaffolds can easily be functionalized, for instance by incorporating active species from the beginning of the process. This has been demonstrated by incorporating enzymes in freeze-cast materials [78], although it is limited to the case where no high temperature consolidation step are used.

Few reports of the biological response of freeze-cast materials can be found, such as pre-osteoblastic cell proliferation *in vitro* in pore channels of 25 and 100 μm [24,31], or the implantation of porous HAP scaffolds in femoral bone cavities of rabbits [31]. Although promising, additional results are required to validate the potential of this new class of material architecture.

Further developments of the process should focus on two key aspects:

- A careful structure/property relationship assessment, which is still lacking today, although interesting progress has been made. Such results will provide the necessary guidelines to adjust the process and tailor the structure to the actual functional requirements.
- *In vitro* and *in vivo* tests to validate the potential of these materials and the various hypotheses related to the structure, such as the allegedly facilitated fluids and cell penetration into the scaffolds arising from the directionality of the structure.
